# Sophia Jex-Blake: A Great Personality

**Published:** 1918-08-03

**Authors:** Mary Scharlieb


### SOPHIA JEX-BLAKE: A GREAT PERSONALITY.

### She Opened the Medical Profession to Women.

THIS beautiful and deeply interesting book has a power which compelled me to read it all through. My duty as Reviewer might have been performed after a more superficial perusal, but the charm of the author, the tremendous personality of the subject, and the significance of the circumstances recorded left me no choice. I had to read it whether I had time to do so or not.

Dr. Margaret Todd writes of Dr. Sophia Jex-Blake* in the spirit of a hero-worshipper, but she clearly sees and honestly indicates that her hero is a woman and not a goddess. From the very commencement of her turbulent, unmanageable childhood, throughout her stormy career, and up to the close of her long fight we see in Sophia Jex-Blake the conflict of good and evil, the loving heart and the hot temper, the business capacity, and the impulsive unwisdom—the charm which gave her friends and the difficulties of temperament that deprived her of them.

The first six chapters deal with the childhood and the school and college life of this most remarkable woman, "S. J.-B.," as her chronicler affectionately calls her. One can well understand, in the light of her subsequent history, that the young Sophia was "a terrible pickle," that her school life was far from being continuous or smooth, and that she was often too turbulent and impossible to be compatible with the welfare of a somewhat delicate mother.

Indeed, one of the interesting features of the book is the story of the gradual development in the maternal and filial relationships between Mrs. Jex-Blake and her loving, gifted, but wayward child. Love there always was on both sides, but the development of mutual understanding, progressing into ideal sympathy and the tenderest devotion, was the work of many years. The wonder is how Mrs. Jex-Blake, early Victorian in outlook and narrowly Evangelical in creed, could gradually expand until she became the perfect companion and most intelligent sympathiser with her daughter, whose gifts and career might have seemed likely to alienate one with so totally different an outlook and experience of life.

Miss Jex-Blake's spiritual relationship with her father matured more slowly, but it progressed towards a deep mutual respect founded on a more than usually tender filial love. Indeed, the conquest of her parents and her family, and their conversion to thorough approval of her life-work in opening the medical profession to women, is an eloquent testimony to the justice of her aims and to the ability with which she overcame the great difficulties which confronted her.

Queen's College, in Harley Street, has the honour of being Sophia Jex-Blake's college, and of having had her services as mathematical tutor. In connection with this episode Dr. Todd tells us of the contest between the young tutor and her father. He was willing for her to work, but most unwilling that she should receive payment. He thought that a lady of independent means disgraced herself and her family if she accepted any pecuniary acknowledgment.

Next, in Chapter VII. comes the brief and pathetic story of the friendship between Miss Jex-Blake and Miss Octavia Hill. Affection seems to have been deep and true on both sides, but in this matter, as in many others in her life, Sophia Jex-Blake seems to have been incapable of adapting herself to other people, and even incapable of understanding how and why she hurt or offended them. Apparently the love that existed between these two remarkable women survived throughout life, although they never met again and their intercourse was limited to the exchange of appreciative letters at long intervals.

In all lives that are to be of great service to the world we may trace the strengthening, refining, and educative value of their disciplinary environment. Miss Jex-Blake could not have lived her very difficult life and brought her apparently hopeless task to a triumphant issue had she not first graduated in the hard school of painful and varied experiences. Her stormy childhood, difficult school-days, the perplexities at Queen's College, and her sorrow in her broken friendship were all phases of her necessary discipline. To them succeeded a time in Edinburgh marked by educational struggles and religious difficulties. During this period she was first brought into contact with Miss Elizabeth Garrett, afterwards Mrs. Garrett-Anderson. Miss Garrett had been trying to induce the London University to open its medical degrees to women. This effort had failed for the time and she accepted Miss Jex-Blake's invitation to see what could be done in Edinburgh. Here, too, nothing immediate was accomplished, and, indeed, a whole generation was to go by and an enormous expenditure of money, effort, and pain had to be encountered before the University of Edinburgh opened its doors to women in 1895.

At this time Sophia Jex-Blake had no desire for a doctor's life. Her ambition was to be a good teacher, and in furtherance of this object her next move was to Germany, where, for a season, she underwent the discipline of exile and loneliness as a teacher at Mannheim. As usual there was a want of mutual comprehension between her and her colleagues, and she was thoroughly worn and weary before the time came to return to her home and to her mother's ever potent and welcome sympathy.

In 1865 Miss Jex-Blake paid a visit to America, which was fated to be the turning point of her life. She wrote in her diary "I have such a feeling that with the new world a new life will open," and so it was. She met Dr. Lucy Sewall, a most charming young woman doctor, whose gifts and

* *The Life of Sophia Jex-Blake.* By Margaret Todd, M.D. (London: Macmillan & Co. 18s. net.)

**The Life of Sophia Jex-Blake—(*continued*).**

grace were complementary to her own. The admiration and respect which she felt for Dr. Sewall were mutual and formed the basis of a most happy friendship which lasted until death.

From this time began Miss Jex-Blake's life-work—the arduous struggle to obtain for women opportunities of medical study and the possibility of qualification. The difficulties of this enterprise were so great that it is rare to find anyone—even among the medical women themselves—who adequately appreciates the debt she owes to Sophia Jex-Blake. Ignorance and custom blinded the public to the need for women doctors, the medical profession, in general, could not see any reason for their existence or any way of permitting them to study and to qualify; indeed, all their instincts, their interests, and even their better feelings united in making them think that women were utterly unsuitable for the duties of doctors. In addition to these formidable outside deterrents, Miss Jex-Blake's health was never robust, and her temperament was distinctly unsuited to a task which called for tact, discretion, and humility, which she lacked, quite as much as for the courage, tenacity, and business capacity with which she was richly endowed.

The history of the endless efforts, hopes, fears, disappointments, and temporary failure of Miss Jex-Blake and her six colleagues to secure admission to the medical degrees of the University of Edinburgh has been often told. The drama occupied the stage for twenty-five long and exhausting years, and, strange to say, left its chief actor worn and weary indeed, but neither embittered nor indifferent. To the very last she believed in the men who had helped her so generously, and she forgave those who had deceived and betrayed her cause. Miss Jex-Blake was cast in a heroic mould—there was nothing small or petty about her. The fact that the School of Medicine for Women in London founded by her preferred to elect another lady as its first Honorary Secretary failed to alienate her kindly interest in its fortunes.

Truly, there were giants in the earth in those days! Mary Scharlieb.

